# The impact of frailty on the management of atrial fibrillation

**DOI:** 10.18632/aging.204918

**Published:** 2023-07-19

**Authors:** Stephanie L. Harrison, Søren P. Johnsen, Gregory Y.H. Lip

**Affiliations:** 1 Liverpool Centre for Cardiovascular Science at University of Liverpool, Liverpool John Moores University and Liverpool Heart and Chest Hospital, Liverpool, United Kingdom; 2 Department of Cardiovascular and Metabolic Medicine, Institute of Life Course and Medical Sciences, Liverpool, United Kingdom; 3 Danish Center for Clinical Health Services Research, Department of Clinical Medicine, Aalborg University, Aalborg, Denmark

**Keywords:** frailty, atrial fibrillation, multimorbidity and polypharmacy, ABC pathway

Atrial fibrillation (AF) increases in prevalence with age and patients with AF often have multiple cardiovascular and cardiovascular-related co-morbidities. Although age is a well-established risk factor for incident cardiovascular disease or worse outcomes with cardiovascular disease, there is marked heterogeneity amongst people of the same age. The concept of frailty was established to identify older people who have an accumulation of deficits. There is also alignment of frailty with ageing, multimorbidity and polypharmacy, resulting in a ‘clinically complex’ group of patients.

The impact of frailty on outcomes has not been previously well characterised in populations with AF. A previous cross-sectional analysis of 1,244 patients with nonvalvular AF showed 14% were classified as frail [1]. The characteristics of the population with AF and frailty were described in this study but given the cross-sectional nature of this analysis, outcomes for people with AF by frailty status could not be examined.

In a recent study using the European Society of Cardiology (ESC)-European Heart Rhythm Association (EHRA) EURObservational Research Programme (EORP) AF General Long-Term Registry, the concept of frailty was further examined in relation to outcomes in 10,177 individuals with AF [2]. In the ESC-EHRA EORP-AF General Long-Term Registry, more than one in five individuals in the population were defined as frail (21.3%) following evaluation by the 40-Item Frailty Index derived from the cumulative deficits model. Increasing frailty was associated with both higher risk of stroke as measured with the CHA_2_DS_2_-VASc score and higher risk of bleeding as measured with the HAS-BLED score. Furthermore, frailty was associated with higher risk of adverse outcomes over a mean follow-up period of 1.84 years, including cardiovascular, non-cardiovascular and all-cause mortality, major adverse cardiovascular events as a composite outcome and major bleeding.

Whilst the concept of frailty has been well-established, the applicability of pre-frailty is less clear. In the ESC-EHRA EORP-AF General Long-Term Registry, 59.6% of the population were identified as pre-frail [2]; however, frailty may not need to be progressive in all patients and frailty is more likely to be modifiable if detected at an earlier stage [3]. Therefore, identification of pre-frailty may have important implications for risk factor reduction and optimising management for patients with AF.

The impact of frailty on clinical decision making for managing patients with AF such as the prescription of oral anticoagulants (OACs) is unclear. A cross-sectional analysis completed in the ESC-EHRA EORP-AF General Long-Term Registry examined frailty status and clinical management at baseline discharge and showed that frailty was associated with lower odds of being prescribed OACs, compared to patients without frailty (Odds Ratio 0.70, 95% confidence interval 0.55-0.89) [2]. Conversely, a registry study of 14,493 older individuals with AF living in care homes in Wales demonstrated that advancing levels of frailty based on the electronic Frailty Index (eFI) which is collected in primary care practices was associated with higher odds of being prescribed OACs (Odds Ratio 4.61, 95% confidence interval 3.95-5.38 for people with mild frailty vs. no frailty) [4]. Nonetheless, a previous cross-sectional analysis reported no significant association between frailty status and OACs prescribing; however, the sample size was markedly smaller (1,244 patients) and a biological model of frailty based on only five components was used [1]. The pattern of less OAC being prescribed with increasing clinical complexity factors (despite their higher risk of clinical events) is evident from other cohorts [5].

Differences in the study population and the tool used to assess frailty may explain differences in study findings when examining the association between frailty and prescription of OACs. Furthermore, the timing of when the studies were conducted may have influenced the findings. Non-vitamin K antagonist OACs (NOACs) were first introduced in the early 2010s and international guidelines have been updated to recommend the use of NOACs as first-line treatment to reduce the risk of stroke in patients with AF [6]. Indeed, a large registry-based study including >700,000 patients with AF mainly based in the United States demonstrated a substantial increase in the proportion of people aged ≥80 years receiving OAC from 32.4% in 2011 to 43.6% in 2019 [7].

Frailty alone should not be reason to withhold oral anticoagulation in patients with AF. In patients with frailty, the benefits of oral anticoagulation outweigh the small absolute risk of bleeding [6]. The latter risk can be carefully assessed, and mitigated by attention to modifiable bleeding risk factors and regular follow-up. The majority of previous randomised controlled trials examining the use of NOACs for patients with AF have had a mean age of approximately 70 years and excluded individuals with serious co-morbidities or with a life expectancy of <2 years. However, the recent ELDERCARE-AF trial examined the use of low-dose edoxaban compared to placebo in patients with AF ≥80 years, including 41% of patients identified as frail, showing that low-dose edoxaban was superior in preventing stroke and systemic embolism, with no significantly higher incidence of major bleeding or intracranial haemorrhage (Okumura, N Engl J Med, 2020). Large-scale observational data of >300,000 individuals aged ≥80 years has also suggested the use of NOACs in this population was associated with improved outcomes including lower risk of dementia, ischaemic stroke and all-cause mortality compared to no OAC, with the exception of a small but statistically significant higher risk of major bleeding [7].

**Figure 1:**
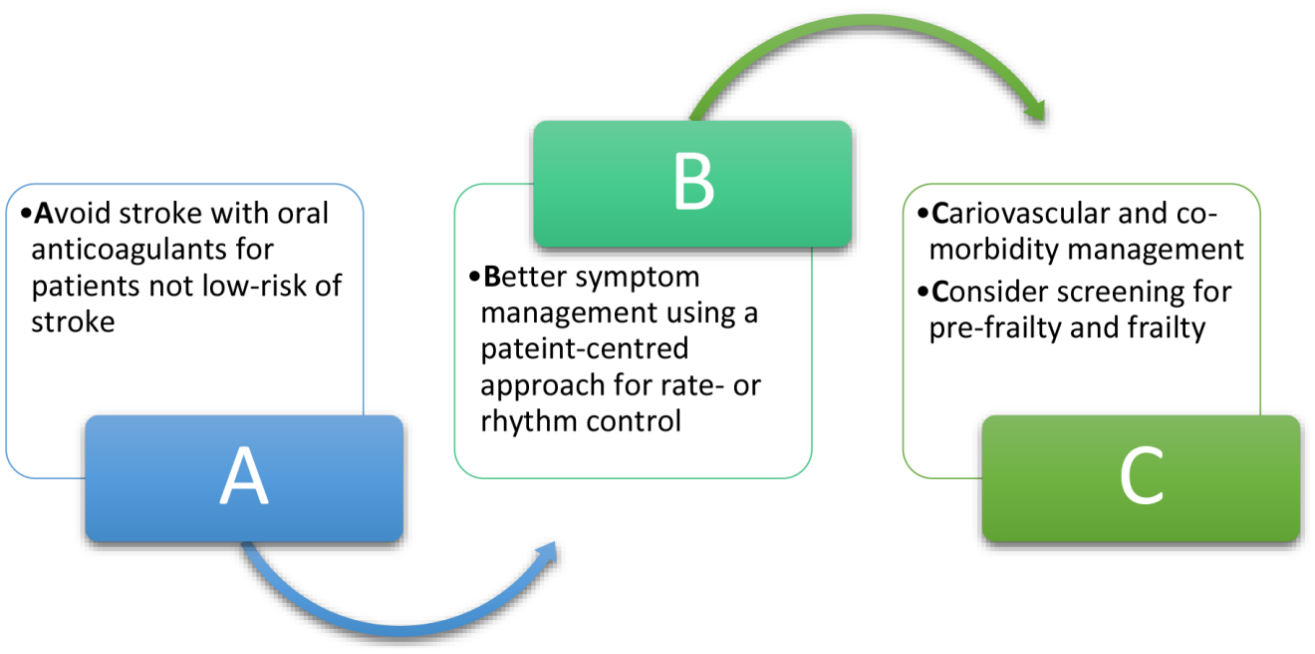
Extension of the Atrial Fibrillation Better Care (ABC) pathway to include consideration of screening for pre-frailty and frailty in the “C” component of the pathway.

The Atrial Fibrillation Better Care (ABC) pathway was developed as a holistic approach to the management of patients with AF and is described in guidelines for the management of AF [6]. The ABC pathway is recommended in clinical guidelines and includes three pillars: A) avoid stroke with the use of oral anticoagulants, B) better symptom management with rate- or rhythm control and C) cardiovascular and other co-morbidity management. Although frailty is not described in the ABC pathway per se, assessment of frailty or pre-frailty could be incorporated into part “C” of this pathway to determine if there are further potentially modifiable risk factors which could be optimised to reduce the risk of frailty progression and optimise outcomes for patients with AF (Figure 1). Indeed, this is the basis of the Horizon Europe-funded multinational ‘Atrial fibrillation integrated approach in frail, multimorbid and polymedicated older people’ (AFFIRMO) Programme [8]. This consortium will address the role of multimorbidity in AF patients and to validate the effectiveness of patient-centred, stratified management for older AF patients with multimorbidity using the ABC pathway. Another parallel Horizon Europe funded project, EHRA-PATHS, is also currently developing and evaluating care pathways to tackle multimorbidity, polypharmacy and sex differences in elderly AF patients (Heidbuchel, European Heart Journal, 2022).

Further contemporary research is needed to understand how frailty impacts clinical decision making for the management of AF. There are many tools available to measure frailty and it is unclear if the current tools used to measure frailty in clinical practice are sufficient for differentiating older patients with AF at highest-risk of outcomes. Assessment of pre-frailty in patients with AF and the development of strategies to address potentially modifiable components of pre-frailty to reduce the risk of frailty progression could have important implications to optimise care.
